# Helping to prioritise interventions for depression and schizophrenia: use of Population Impact Measures

**DOI:** 10.1186/1745-0179-2-3

**Published:** 2006-03-22

**Authors:** Richard F Heller, Islay Gemmell, Lesley Patterson

**Affiliations:** 1 Evidence for Population Health Unit, Division of Epidemiology and Health Sciences, University of Manchester, Manchester M13 9PT, UK

## Abstract

**Background:**

To demonstrate the potential of Population Impact Measures in helping to prioritise alternative interventions for psychiatry, this paper estimates the number of relapses and hospital readmissions prevented for depression and schizophrenia by adopting best practice recommendations. The results are designed to relate to particular local populations.

**Methods:**

Literature-based estimates of disease prevalence, relapse and re-admission rates, current and best practice treatment rates, levels of adherence with interventions and relative risk reduction associated with different interventions were obtained and calculations made of the Number of Events Prevented in your Population (NEPP).

**Results:**

In a notional population of 100,000 adults, going from current to 'best' practice for different interventions, the number of relapses prevented in the next year for schizophrenia were 6 (increasing adherence to medication), 23 (family intervention), 43 (relapse prevention), and 44 (early intervention); and for depression the number of relapses prevented in the next year were 100 (increasing care management), 227 (continuing treatment with antidepressants), 279 (increasing rate of diagnosis), and 325 (Cognitive Behaviour Therapy). Hospital re-admissions prevented in the next year for schizophrenia were 6 (increasing adherence to medication), 36 (relapse prevention) and 40 (early intervention).

**Conclusion:**

Population Impact measures provide the possibility for a policy-maker to see the impact of a new intervention on the population as a whole, and to compare alternative interventions to best improve psychiatric disease outcomes. The methods are much simpler than others, and have the advantage of being transparent.

## Background

There are a number of potential interventions in psychiatry that would help improve patient outcomes. The problem is how to decide which ones to prioritise for service development. While measures, such as Number Needed to Treat (NNT) and Quality Adjusted Life Years (QALY), do provide estimates of the benefit of interventions, they do not allow impact at the population level to be quantified. We have developed a new set of Population Impact Measures (PIMs) to describe the population impact of risks and benefits [1-4]. Population Impact Measures are simple to compute, and contain the elements to which policy-makers would have to pay attention in the commissioning or improvement of services. For describing the population impact of an intervention, the Number of Events Prevented in a Population (NEPP)[2] describes the impact of treatment or other interventions and is defined as *"the number of events prevented by the intervention in your population over a defined time period"*.

The measure NEPP is a population extension of the well-known Number Needed to Treat (NNT), and takes into account the frequency of the condition in the population and the proportion of those with the condition who are actually exposed to the intervention. The measure provides local context to previous measures, allowing policy-makers to identify and prioritise the potential benefits of interventions on their own population. This paper derives NEPP for interventions used in two important psychiatric conditions, and demonstrates the potential for helping to prioritise interventions to make maximal impact on the population.

## Methods

We have obtained literature-based estimates of the components required to calculate the NEPP for a number of different interventions for schizophrenia and for depression. There are a number of relevant practice guidelines in the UK [5-7], and in the US [8,9], but none of them give an idea of the benefit to a particular population. The NEPP can be used to estimate the incremental impact of moving from current to best practice and is calculated as shown in Table 1.

**Table 1:** Formula for calculating Number of Events Prevented in your Population (NEPP)

*NEPP *= n * *P*_d _* *P*_e _* *r*_u _* *RRR * where
*n *= population size
*P*_*d *_= the prevalence of the disease in the population
*P*_*e *_= the proportion eligible for treatment
*r*_*u *_= the risk of the event of interest in the untreated group or baseline risk
*RRR *= the relative risk reduction associated with the treatment

In order to reflect the incremental effect of changing from current to 'best' practice and to adjust for levels of compliance the proportion eligible for treatment *P*_*e *_is ((*P*_*b *_- *P*_*t*_) * *P*_*c *_where *P*_*t *_is the proportion currently treated, *P*_*b *_is the proportion that would be treated if best practice was adopted and *P*_*c *_is the proportion of the population who are compliant with (adherent to) their medication.

We chose to examine the population impact of interventions recommended in the NICE guidelines for depression [10] and schizophrenia [11] wherever we could identify data to make the calculations, for a notional population of 100,000 adults. Thus for depression, we examined continuing treatment with antidepressant therapy, screening to increase the rate of diagnosis, and cognitive behaviour therapy (CBT). We also included increasing care management as there are a number of relevant trials. For schizophrenia, we examined intervention by an early intervention team, increasing adherence to drug treatment, and family and relapse prevention interventions. We were unable to find appropriate data for CBT, assertive outreach, crisis resolution or home treatment teams. We chose hospitalisation and relapse as outcomes with different perspectives, although since hospitalisation is rare in depression and not used in most trials, we restricted the use of this outcome to schizophrenia. Table 2 lists the interventions which we included, and those we excluded, in our analysis.

**Table 2:** Interventions included in the NICE recommendations which were included (or excluded) in this analysis

Depression:
• Continuing treatment with antidepressant therapy
• Screening to increase the rate of diagnosis
• Cognitive behaviour therapy (CBT)
• Increasing care management (not included in NICE recommendations, but evidence found)
Schizophrenia
• Early intervention
• Increasing adherence to drug treatment
• Family intervention
• Relapse prevention

CBT, assertive outreach, crisis resolution or home treatment teams not included as no evidence for benefit found

The data on prevalence and evidence on effectiveness come from a review of a number of papers, some are systematic reviews and some are individual trials. Where differences in estimates are found between publications, an estimate that represents the consensus has been attempted, using median or mean values depending on the data.

Since we have had to use estimates for many of the variables, we have performed a one-way sensitivity analysis [12,13] to explore the differences that would have been seen with the use of different estimates. We calculated the NEPP for the minimum and maximum of each parameter while holding the other parameters constant. For the relative risk reduction we used the lower and upper limits of the 95% confidence interval as the minimum and maximum estimates. We used the minimum of the minimum estimates and the maximum of the maximum estimates as our interval of plausible values for the NEPP.

## Results

### Depression

The prevalence of major depression was taken from Kessler [14] at 6.6%, although estimates ranged from 5% [15] to 8%[13]. Since only 48% of patients currently have their depression diagnosed (taken from 45%[13] and 51% [14], we have adjusted the prevalence estimates to those currently diagnosed, and used the converse for the estimates of the prevalence of undiagnosed depression. The outcome of 'relapse' was used (1-year wherever possible), since hospitalisation is rare in this condition, and most trials did not report it. Where relapse was not used, but 'remission' reported, the converse of remission was assumed to represent relapse. The baseline risk of relapse ranged from 41%[16] to 65%[13], and we took 50% as our estimate. The proportion of the population currently receiving each intervention was obtained from various sources. We assumed that 50% of patients are currently receiving continuing treatment with anti-depressants, and only 5% currently have increased care management and Cognitive Behaviour Therapy (CBT). Best practice intervention goals are assumed to be 75% for each intervention. Compliance/adherence with each intervention is taken as 82% for continuing treatment with anti-depressants[16], and 60% (from 66% [17] and 51% [18] for increasing care management. We have used 58% for compliance with CBT, based on a summary of the various data sources we could find [19-21].

For increasing the diagnosis of depression, we assumed that we would identify 84% of those previously undiagnosed (being the sensitivity of the screening instrument[13] and that 63% would be compliant with the medication started as a result of the screening diagnosis [22]. Relative Risk Reduction is taken as 70% for continuing treatment with antidepressants[16], 41% for increasing diagnosis (from relative benefits of 1.6 and 1.7 for pharmacotherapy in comparison with placebo [23], 15% increasing care management (taken from Odds Ratios of 1.9 for remission [24], and 50% for CBT (50% relapse rate reduction compared with antidepressants [25] and 66% in recurrent depression [26]). These data are summarised in Table 3.

**Table 3:** Depression: data used for the calculations. Figures are percentages (range).

Treatment	Continuing treatment with antidepressants	Increasing rate of diagnosis	Increasing care management	Cognitive Behaviour Therapy
Prevalence of depression	3.2* (2.6,3.8)	3.4** (2.9,4.2)	3.2* (2.6,3.8)	3.2* (2.6,3.8)
Outcome	Relapse	Relapse	Relapse	Relapse
Baseline risk of outcome (relapse) in next year	50 (41,65)	50 (41,65)	50 (41,65)	50 (41,65)
Percentage of those with the disease currently receiving treatment (or diagnosis)	50	0	5	5
'Best practice' treatment (or diagnosis) goal	75	75	75	75
Compliance with treatment (or diagnosis)	82 (30,97)	84x63	60 (51,66)	58 (47,79)
Relative Risk Reduction associated with the treatment	70 (62,78)	41 (23,56)	15 (1,27)	50 (34,64)
Numbers of Events Prevented in the Population, and (95% Confidence Intervals), by each intervention in the next year among a population of 100,000, going from current to 'best' practice	227 (83,295)	279 (157,381)	100 (7,180)	325 (219,442)

* prevalence (%) currently diagnosed

** prevalence (%) currently un-diagnosed

### Schizophrenia

Prevalence is estimated as 3/1000 population, consistent with data from three reports [27-29], although this includes a variety of clinical states and will vary according to demographic variables. We have used two different outcomes, hospitalisation and relapse, both over 1-year or as close to it as possible. Relapse is defined differently in different studies [30-33], ranged from 44% to 50%, and we have used 48%. Hospitalisation ranged from 39% [34] to 50%[30] and 51% [33], and we have used 45%. The proportion currently receiving the intervention was assumed to be 0 for each of the interventions, with the exception of increasing adherence to medication, for which 66% was the current baseline adherence rate in the intervention arm of trials to increase adherence [35]. The best practice goal for each intervention was estimated to be 75%, apart from increasing adherence to medication, where 76% were adherent after the intervention [35]. Adherence to the intervention was taken from the intervention arm of the trials or summaries of trials, and was 76% for early intervention [33], and estimated as 80% for the other interventions (although not relevant for the increasing adherence to medication intervention where adherence was already taken into account in estimating the best practice goal). Relative Risk Reductions for relapse and hospitalisation respectively were 54% and 52% for early intervention [33], 50% and 44% for relapse prevention [34] and 28% for relapse only for family intervention [31]. Relative Risk Reduction for increasing adherence was estimated as 45%: 64% (Odds Ratio of 2.8 [36] for increasing medication adherence) multiplied by the effect of the medication itself estimated at 70% reduction in relapse rate (and applied also to re-hospitalisation)[37]. These data are summarised in Table 4.

**Table 4:** Schizophrenia: data used for the calculations. Figures are percentages (range)

Treatment	Early Intervention Team		Increase adherence to medication		Family intervention		Relapse prevention	
Prevalence	0.3 (2.3,7.4)		0.3 (2.3,7.4)		0.3 (2.3,7.4)		0.3 (2.3,7.4)	

Outcome	Relapse	Hospital re-admission	Relapse	Hospital re-admission	Relapse	Hospital re-admission	Relapse	Hospital re-admission
Baseline risk of outcome in next year	48 (44,50)	45 (39,51)	48 (44,50)	45 (39,51)	48 (44,50)	45 (39,51)	48 (44,50)	45 (39,51)
Percentage of those with the disease currently receiving treatment	0	0	66*	66*	0	0	0	0
'Best practice' treatment goal	75	75	76	76	75	75	75	75
Compliant with treatment	76	76	N/A	N/A	80	80	80	80
Relative Risk Reduction associated with the treatment	54 (3,78)	52 (3,78)	45 (36,49)	45 (36,49)	28 (15,40)	N/A	50 (-7,77)	44 (-10,72)
Numbers of Events Prevented in the Population, and (95% Confidence Intervals), by each intervention in the next year among a population of 100,000, going from current to 'best' practice	44 (2,109)	40 (2,98)	6 (5,16)	6 (5,15)	23 (13,59)	N/A	43 (-6,106)	36 (-8,87)

* currently adherent to medication

### Number of events prevented

The NEPP for relapse for both depression and schizophrenia and hospital re-admission for schizophrenia are also shown in Tables 3 and 4. In a notional population of 100,000 adults, going from current to 'best' practice for different interventions, the number of relapses prevented in the next year for schizophrenia were 6 (increasing adherence to medication), 23 (family intervention), 43 (relapse prevention), and 44 (early intervention); and for depression the number of relapses prevented in the next year were 100 (increasing care management), 227 (continuing treatment with antidepressants), 279 (increasing rate of diagnosis), and 325 (Cognitive Behaviour Therapy). Hospital re-admissions prevented in the next year for schizophrenia were 6 (increasing adherence to medication), 36 (relapse prevention) and 40 (early intervention).

They demonstrate a large difference in relapses prevented between depression and schizophrenia, and smaller differences between the interventions for each condition.

## Discussion

The choice of conditions to examine in this work was dictated by those interventions for which evidence of benefit was available from a literature review. Some of the interventions included in guideline recommendations do not meet this criterion. In the case of CBT in schizophrenia, a systematic review showed no effect on relapse or re-admission[38], although some individual trials have shown an effect [39]. A review of trials of crisis intervention did show an effect, but this was dependent on only one trial [40]. Where possible, we used systematic reviews, since individual trials often provide conflicting information (as in attempts to increase compliance with therapy [36,41,42]).

One of the most difficult issues in interpreting the results of, and comparing between interventions, in the field of psychiatry, is the decision about which outcome measure to use [43]. Interventions for different diseases or conditions will have different outcomes. Some may result in an improvement in mortality, others in morbidity. This has led to the creation of generic outcome measures, such as the Quality Adjusted Life Year (QALY) and the Disability Adjusted Life Year (DALY), which have the advantage of allowing direct comparison of outcome between different interventions. They rely on derivation of weights and values for different disease outcomes, which have been criticised as being arbitrary, although they have been used extensively in assessments of the cost-effectiveness of interventions in mental health[28,44-46]. The advantage of our measures is that they allow the policy-maker to see exactly which outcomes are being affected. For example, a hospital manager might prioritise the prevention of hospital re-admissions, while a patient or carer might be more interested in symptom relapse. The use of various measures depends on which ones have been included in the studies contained in the evidence base, which vary between study and condition.

Use of Population Impact Measures (PIMs), allows a population perspective to the estimate of risks and benefits. If an intervention is effective in individuals, but the condition it is used to treat is uncommon in your own population, its impact on the population will be more limited than an alternative intervention with lower effectiveness but which can be used on a larger proportion of the population. The findings here demonstrate the different population impact of interventions for conditions of different prevalence in the population. We see from Tables 3 and 4, that the numbers of relapses prevented by each of the interventions for depression are much higher than those for schizophrenia. The differences in population impact between interventions for depression and schizophrenia reflect the difference in prevalence between the two, consistent with estimates by Andrews[44]. Whether there is a real choice to be made between depression and schizophrenia is debatable, since, at least in the UK, the responsibility for those in the population with depression is mainly shouldered by primary care whereas specialist psychiatric services are the agencies mostly responsible for delivery of care for people with schizophrenia[47]. It is also difficult to compare relapse in the two conditions. Since hospitalisation is rare in depression, we cannot compare hospitalisations between the two conditions. Within-condition comparisons between different interventions may allow the policy-maker to prioritise between them. We see large differences in population impact between different interventions although the range of impact indicated by the sensitivity analysis demonstrates how susceptible these measures are to variation in the parameter estimates on which they are based.

Another advantage of the use of Population Impact Measures, is that they provide an estimate in a particular population, of going from current to 'best' practice. The numbers are thus dependent on current practice levels and the treatment goals. Where the goals are close to current practice, the potential population gain will be lower than where the gap is large, due either to poor current practice or ambitious goals. The potential impact thus can be personalised to the individual population into which the intervention is to be introduced. The results we present will thus vary according to local settings. Although the data used here come from a UK setting, the method is fully transportable to other populations.

Although cost data would be needed to make a final prioritisation decision, we have deliberately left the outcomes to be expressed in the terms in which the data were collected (rather than the generic measures used in cost-effectiveness estimates) so that benefit expressed as real outcomes can be identified. A subsequent step would be to prioritise between interventions, taking costs into account, once the population impact can be observed.

The estimates we have made depend on the availability of relevant data. We have used literature-based estimates, which in themselves vary considerably. For example, adherence to drug treatment for depression varied from 30 to 97% in a number of trials, with a median of 63% [22]. We have used a different value for adherence to *continuing *drug treatment for depression (82%), based on the non-withdrawal rate from trials in a systematic review[16]. The estimates used in this paper for adherence, both to medication and to adherence enhancing interventions, may thus not relate to local practice settings. Similarly, access to non-pharmaceutical interventions will vary considerably. Depending on the definition used, in the UK, there may be 20 times more CBT practitioners per population in the best provided 10% of the population than in the worst 10%, and our estimate of 5% of current exposure to CBT for depression is greater than the 1% national estimate of availability to those who might benefit [48]. The accuracy of the estimates would be increased by better availability of local data from the population to which they would be applied, and the gaps in the data necessary for the preparation of this paper demonstrate a clear need for better relevant data. However, it should be noted that local data may be plagued by small numbers causing under-ascertainment of prevalence and outcomes. The need for good local data should motivate improvements in local data collection systems. If good local data were available, the variability in the NEPP would only be due to variability in measures of risk and RRR and where good meta-analyses exist the RRR estimate is relatively stable. The method could also be adapted to explore the benefits of policy changes such as those designed to prevent suicide, however, this will depend on obtaining some estimate of benefit. A previous paper estimated population attributable fractions for alternative strategies for preventing suicide, and recommended population-based rather than high-risk strategies as a result [49]. This methodology is a fore-runner to ours, which allows 'personalisation' to the local population into which the strategy is to be introduced. We have not explored the impact of different population demographic structures or severity case-mix in this paper, but the implications for a local population will depend on the features that determine prevalence, treatment patterns and baseline risk of disease.

## Conclusion

The use of Population Impact Measures provides the possibility for a policy-maker to see the impact of a new intervention on the population as a whole and to compare alternative interventions. Our methods are much simpler than those which use generic outcomes such as the QALY [28,44,45], and have the advantage of being transparent. We believe that not only will their use assist in priority setting, but that their transparency will allow the policy-maker to identify gaps in the availability of local (and trial) data – information which is essential if an evidence base is to be used to prioritise interventions based on improving the health of the population [50].

## Competing interests

The author(s) declare that they have no competing interests.

## Authors' contributions

RFH conceived the study, RFH and LP reviewed the literature, IG performed the calculations. All were involved in the drafting the manuscript, and have read and approved the final manuscript.
